# Mediastinal malignant triton tumor: A rare case series and review of literature

**DOI:** 10.1016/j.ijscr.2019.08.020

**Published:** 2019-08-20

**Authors:** Ikram Chaudhry, Thabet Algazal, Ahsan Cheema, Aman Al Faraj, Noor Al Malki, Hadi Mutairi, Ahmed Abbas, Samir Amr

**Affiliations:** Department of Thoracic Surgery and Pathology, King Fahad Specialist Hospital Dammam, Saudi Arabia

**Keywords:** Mediastinum, Malignant triton tumor, Surgery, Radiotherapy, Chemotherapy

## Abstract

**Introduction:**

In 1938, the Malignant Triton Tumor (MTT) was first explained by Mason.

**Case presentation:**

Case 1: A man aged 28 years presented with chest pain and difficulty in breathing since last five months, there was no history of cough fever or night sweats. Clinical examination was unremarkable. His routine hematological tests including tumor markers were within normal range, testicular ultrasound was normal. CT scan of thorax revealed a mass in the anterior mediastinum. CT guided biopsy revealed a malignant triton tumor.

Case 2: A 30 years old man, nonsmoker presented with history of chest tightness and feeling pressure while kneeling down since last 3 months, otherwise fit and healthy. His routine hematological investigations including tumor markers were within normal range. A CT scan of thorax revealed a large mass in the right posterior mediastinum. CT guided biopsy showed malignant triton tumor.

Case 3: A man aged 28 years presented with chest pain and difficulty in breathing since last five months, there was no history of cough fever or night sweats. Clinical examination was unremarkable. His routine hematological tests including tumor markers were within normal range, testicular ultrasound was normal. CT scan of thorax revealed a mass in the anterior mediastinum. CT guided biopsy revealed a malignant triton tumor.

**Discussion:**

Malignant peripheral nerve sheath tumors (MPNST) are uncommon sarcomatous tumors that are believed to be derived from Schwann cell or neighboring cells with perineurial differentiation. MTT is rarely reported in mediastinum, lung and heart (<10%) To the best of our knowledge, only few cases of MTT in the mediastinum have been reported in English literature, including, four were reported in the anterior mediastinum, three in the posterior mediastinum, one in the middle mediastinum and one between the ascending aorta and the main pulmonary artery. Most of the patients were young adults. We report three cases of rare mediastinal malignant triton tumors. They have been treated with palliative surgery/radical surgery +/− adjuvant therapy. The prognosis varied from a 3 month overall survival time to being alive at a 53 month follow-up period.

**Conclusion:**

In conclusion we report three rare cases of mediastinal malignant triton tumor treated with radical surgical resection and post-operative radiotherapy, one patient developed lung metastasis, and two had late local recurrence. The malignant triton tumor is a lethal neoplasm which carries very poor prognosis particularly when they occur in the mediastinum because it’s very difficult to obtain wider tumor free margin due to the nature of location site.

## Highlights

• Malignant triton tumor (MTT) is extremely rare subset of malignant peripheral nerve sheath tumor (MPNST) which accounts for < 10% of all MPNST.
• Due to their aggressive biological behavior prognosis is very poor.
• To date in medical literature only 12 cases of malignant triton tumor has been reported.
• We report series of three mediastinal malignant triton.
• Managed with radical surgical resection and neoadjuvent chemotherapy &radiotherapy.

## 1 Introduction

In 1938, the Malignant Triton Tumor (MTT) was first explained by Mason. But, the credit for the introduction of the term ‘malignant triton tumor’ was credited to Woodruff in 1973 [1]. Malignant triton tumor (MTT) is extremely rare subset of malignant peripheral nerve sheath tumor (MPNST) which accounts for <10% of all MPNST, it commonly occurs in young population and 50–70% among those are with Neurofibromatosis Type 1 (NF1) disease [2,3]. Sporadic cases 20–30% has been reported in older age group without NF1. The tumors arising from a peripheral nerve or pre exiting nerve sheath tumor such as neurofibroma are all classified by WHO as MPNST [4]. This case series has been reported according to surgical case series criteria [5].

## 2 Case report

### 2.1 Case 1

**Fig. 1:**
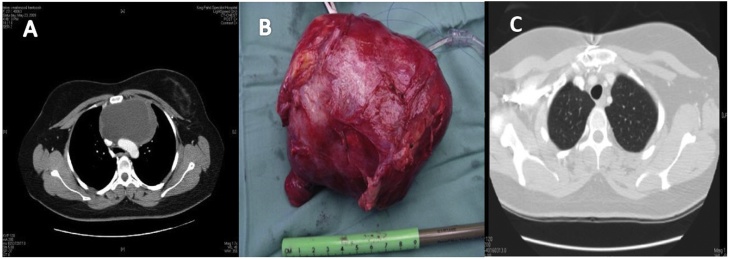
(A) Preoperative CT Scan of thorax showing anterior mediastinal mass (B) Resected specimen (C) Postoperative CT scan of thorax.

A 24 years old man, nonsmoker presents with history of cough and shortness of breath progressively worsening since last six months. There was no history of weight loss or appetite. Routine blood investigations including tumor markers were normal. Computed tomographic scan of chest showed a large anterior mediastinal mass. True cut biopsy revealed malignant triton tumor. Tumor was excised through median sternotomy and post-operative recovery was uneventful. Postoperative Cisplatin 20 mg/m^2^ and Taxanes 135 mg/m^2^ based adjuvant chemotherapy was given. The patient declined adjuvant radiotherapy. He was followed up in outpatient with serial CT scan of thorax and remained disease free for 16 months then he developed local recurrence (Fig. 1(A–C)).

Local recurrence was managed with second line 3 cycles of Doxorubicin 80 mg/m^2^ and Ifosfamide 1.2 g/m^2^. Currently being followed up in the outpatient clinic.

### 2.2 Case 2

**Fig. 2:**
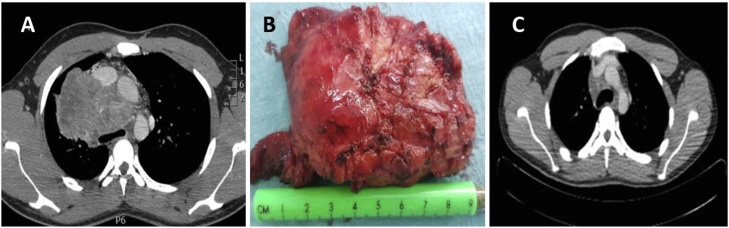
(A) Preoperative CT Scan of thorax showing anterior mediastinal mass (B) Resected specimen (C) Postoperative CT scan of thorax.

A 30 years old man, nonsmoker presented with history of chest tightness and feeling pressure while kneeling down since last 3 months, otherwise fit and healthy. His routine hematological investigations including tumor markers were within normal range. A CT scan of thorax revealed a large mass in the right posterior mediastinum. CT guided biopsy showed malignant triton tumor. Tumor was excised through the right posterolateral thoracotomy and post-operative recovery was uneventful. Postoperative Cisplatin 20 mg/m^2^ and Taxanes 135 mg/m^2^ based adjuvant chemotherapy and radiotherapy 52 Gy, was given. He was followed up in outpatient with serial CT scan of thorax, remained disease free for 24 months then he developed local recurrence (Fig. 2(A–C)).

Local recurrence was managed with second line 3 cycles of Doxorubicin 80 mg/m^2^ and Ifosfamide 1.2 g/m^2^.

Currently being followed up in the outpatient clinic.

### 2.3 Case 3

**Fig. 3:**
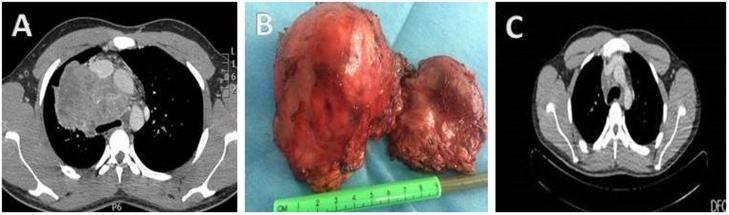
(A) Preoperative CT Scan of thorax showing posterior mediastinal mass (B) Resected specimen (C) Postoperative CT scan of thorax.

A man aged 28 years presented with chest pain and difficulty in breathing since last five months, there was no history of cough fever or night sweats. Clinical examination was unremarkable. His routine hematological tests including tumor markers were within normal range, testicular ultrasound was normal. CT scan of thorax revealed a mass in the anterior mediastinum. CT guided biopsy revealed a malignant triton tumor. Tumor was excised through the median sternotomy. Cisplatin 20 mg/m^2^ and Taxanes 135 mg/m^2^ based adjuvant chemotherapy and radiotherapy 52 Gy, was given. He was followed up in outpatient with serial CT scan of thorax and remained disease free for 17 months then he developed pulmonary metastasis which were resected and histopathology and immune stains confirmed malignant triton tumor (Fig. 3(A–C)).

Pulmonary metastasectomy was performed, the histopathology was consistent with the malignant triton tumor. The patient died six months later.

## 3 Discussion

Malignant peripheral nerve sheath tumors (MPNST) are uncommon sarcomatous tumors that are believed to be derived from Schwann cell or neighboring cells with perineurial differentiation [6]. The incidence of the tumor is rated at one per 100,000, with about 50% of cases occurring in patients with neurofibromatosis type 1 (NF1) [7]. The common targets of MPNST are trunk, head and extremities. However, MPNST can affect many other areas of the body. In rare cases, it has also been reported to occur in the buttock, viscera, retro peritoneum and mediastinum area [8]. A rare and aggressive subgroup of MPNST is known as malignant triton tumor (MTT) which is notable for exhibiting rhabdomyosarcomatous differentiation. The sign of skeletal muscle differentiation can be observed in positive immunohistochemical staining for desmin and myogenin [9]. Many studies have shown that MTT comprises 10% of all Malignant peripheral nerve sheath tumors. Despite that it does not react to chemotherapy and radiation, it is plausible to prolong the life of patients with MTT through the use of surgical resection coupled with adjuvant chemotherapy or radiotherapy. Patients’ 5-year-survival rate is reported to be 14% only [10].

The head, neck and trunk regions are the common targets of MTT. About 20% of MTT cases are reported to be in the head and neck area, 32% of cases are spotted in the trunk region, and 24% of cases are reported to arise in the extremities. MTT is rarely reported in mediastinum, lung and heart (<10%) [11]. To the best of our knowledge, only few cases of MTT in the mediastinum have been reported in English literature, including, four were reported in the anterior mediastinum, three in the posterior mediastinum, one in the middle mediastinum and one between the ascending aorta and the main pulmonary artery. Most of the patients were young adults. They have been treated with palliative surgery/radical surgery +/− adjuvant therapy. The prognosis varied from a 3 month overall survival time to being alive at a 53 month follow-up period. There are two major forms in which MTT can develop; It can either be sporadic or in involvement with NF-1. Between the two forms, the form with association with NF-1 is reported more often compared to the first type. Males are often diagnosed with the NF-1 type of MTT particularly in the younger age group. Females belonging to the older age groups are reported to commonly display the sporadic forms. The progress of the tumor happens following a prolonged latent period of 10–20 years. As per our knowledge only twelve such cases have been reported in the medical literature (Table 1) [[12], [13], [14], [15], [16], [17], [18], [19], [20], [21], [22]].

**Table 1:** To date Cases of Mediastinal Malignant Triton Tumor Reported In English Medical Literature.

Case/year reference	Gender Age year	NF1	Location	Treatment	Recurrence	Follow up
1/1984/12	F/31	YES	Anterior mediastinum	Palliative surgery/radiotherapy	Yes	Overall survival 3 months
2/1984/13	M/29	YES	Posterior mediastinum	No surgery	Yes	Overall survival 6 months
3/1985/14	F/70	NO	mediastinum	Palliative surgery	yes	Alive with disease 53 months
4/1991/15	M/39	NO	Posterior mediastinum	Palliative surgery & chemo radiotherapy	Yes	Overall survival 15 months
5/1996/16	F/17	YES	Anterior mediastinum	Palliative surgery and radiotherapy	Yes	Overall survival 7 months
6/2002/17	M/35	YES	Middle mediastinum	Radical surgery	No	Alive at 18 months
7/2003/18	M/22	NO	Posterior mediastinum	Radical surgery and radiotherapy	No	Alive at 98 months
8/2003/19	M/22	NO	mediastinum	Surgery/radiation therapy	Yes	Survival not reported
9/2006/20	M/30	NO	Anterior mediastinum	Palliative surgery	Yes	Alive at 12 months
				Radical surgery and chemotherapy		
10/2014/21	M/42	NO	Anterior mediastinum	Chemoradiotherap/chines traditional medication	NO	More than one year
11/2018/current	M/28	YES	Anterior mediastinum	Radical surgery	Yes	Overall survival 15 months
12/2018/current	M/32	NO	Middle mediastinum	Radical surgery/radiotherapy	Yes	Overall survival 24 months
13/2018/current	M/45	NO	Posterior mediastinum	Radical surgery /radiotherapy	yes	Overall survival 28 months

Although the origin of triton tumor cells is not certain but based on presence of neural cells and Rhabdomyoblasts some authors hypothesize that two cell components are derived from less differentiated neural crest cells, which can potentially differentiate to mesodermal and ectodermal cells eventually leading to the development of skeletal and neuronal component.

CT and positron emission tomography scans are the imaging tools for the initial diagnosis. PET scan is useful to reveal the distant metastasis in addition to the detail of primary tumor. The final diagnosis is based on the histological and immunohistochemical findings.

**Fig. 4:**
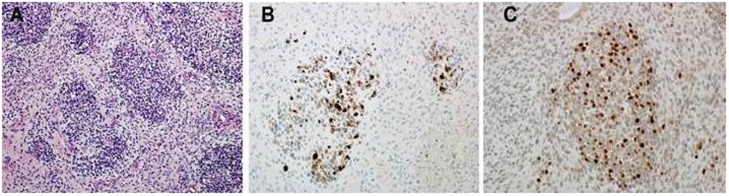
Histopathology Report showed malignant Triton tumor features (A) Higher Magnification featuring several Rhabdomyoblasts with eosinophilic cytoplasm (H&Ex400) (B) Immunostains for desmin show several positive cytoplasmic staining of Rhabdomyoblasts.(C) Immunostains for myogenin show positive nuclear staining of Rhabdomyoblasts.

The diagnosis of MPNST can be established on morphologic grounds as well as S-100 protein and leu-7 positivity (CD57). These tumors display focal S-100 protein positivity at an estimate of 50–90%. This suggests a nerve sheath derivation. However, Rhabdomyoblasts test positive for different types of immunostains namely myogenin, desmin and myo-D1 [23] (Fig. 4).

As established, MTT displays highly-aggressive behavior, thus, having poor prognosis. As a result, the 5-year survival rate of patients affected by the tumor is estimated to be about 14%. McConnell and Giacomantonio reported after reviewing the 124 cases of malignant triton tumor that the overall five years survival rate of 14% and median survival of 13 months and local recurrence rate was 50%.Conversely, patients afflicted with MPNSTs have a 5-year survival rate to range between 16% and 52% [24]. It has been reported that the local recurrence after the tumor resection is common. On the other hand, there have been no further reports of Lymphatic invasion and lymph node involvement in patients diagnosed with MTT. However, the chances of survival of patients have been gradually improved. Radical excision is the standard mode of treatment for the tumors, followed by high-dose radiotherapy. Recent developments in the treatment suggest that neodjuvant therapy and adjuvant chemotherapy can also yield positive results in eliminating micro metastasis. In order to test patients’ responses to therapy, integrated positron emission tomography together with computed tomography have been utilized. There are certain factors that affect the prognosis of MTT such as location, grade, and completeness of surgical margins In particular cases such as when the head, neck and other extremities are affected, prognosis appears to be better [25]. On the other hand, prognosis is worse mediastinum and other affected regions [26]. Compared to one established sporadic forms, related literatures suggest that MTT in association with NF-1 displays worse prognosis [27].

In advanced or metastatic MPNST, outcomes are generally poor. Doxorubicin and ifosfamide are the most effective chemotherapeutic agents [28].

## 4 Conclusion

In conclusion we report three rare cases of mediastinal malignant triton tumor treated with radical surgical resection and post-operative radiotherapy, one patient developed lung metastasis, and two had late local recurrence. The malignant triton tumor is a lethal neoplasm which carries very poor prognosis particularly when they occur in the mediastinum because it’s very difficult to obtain wider tumor free margin due to the nature of location site. In our opinion despite of radical resection with negative margins unexplained biological behavior of such tumor merits further research to achieve further advances regarding the treatment for better survival.

## Funding

There is no funding/grant involved in this case report.

## Ethical approval

Institutional review board (IRB) approval achieved. Ref 018-011 Dated 27/03/2019.

## Consent

A copy of the written consent (for both patients) is available for review by the Editor in Chief of the journal upon request.

## Author contributions

Ikram Ulhaq Chaudhry: Operating surgeon drafting the article, Critical revision and final approval of the article.

Thabet Al gazal MD, Pictures and imaging.

Noor Al Malaki literature review.

Ahsan Cheema, MD Conception and design.

Hadi Al Mutairi Assisted surgery.

Samir Amr MD clinical Pathalogist.

Ahmed Abbas MD Abstract.

## Registration of research studies

UIN 5034.

## Guarantor

The corresponding author Dr Ikram Chaudhry is the guarantor.

## Provenance and peer review

Not commissioned, externally peer-reviewed.

## Declaration of Competing Interest

There is a no conflict of interest in this paper.

## References

[1] Woodruff J.M., Chrink N.L., Smith M.C. (1973). Peripheral nerve sheath tumor with rhabmyosarcomatous differentiation (Malignant Triton tumor). Cancer.

[2] Alina B., Sebastian J.A., Gerardo C. (2015). Malignant Triton tumors in sisters with clinical neurofibromatosis type 1. Case Rep. Oncol. Med..

[3] McConnell Y.J., Giacomantonio C.A. (2012). Malignant triton tumors—complete surgical resection and adjuvant radiotherapy associated with improved survival. J. Surg. Oncol..

[4] Scheithauer B.W., Louis D.N. (2007). Malignant peripheral nerve sheath tumor (MPNST). WHO Classification of Tumors the Central Nervous System.

[5] Agha R.A., Borrelli M.R., Farwana R. (2018). The process 2018 statement: updating conses preferred reporting of case series in surgery (process) guidelines. Int. J. Surg..

[6] Rodriguez F.J., Folpe A.L., Giannini C. (2012). Pathology of peripheral nerve sheath tumors: diagnostic overview and update on selected diagnostic problems. Acta Neuropathol..

[7] Farid M., Demicco E.G., Garcia R. (2014). Malignant peripheral nerve sheath tumors. Oncologist.

[8] Ren W., Xu X., Yan J. (2014). Malignant triton tumor of the anterior mediastinum: a case report. Oncol. Lett..

[9] Kurtkaya-Yapicier O., Scheithauer B.W., Woodruff J.M. (2003). Schwannoma with rhabdomyoblastic differentiation: a unique variant of malignant triton tumor. Am. J. Surg. Pathol..

[10] A1lina B., Sebastian J.A., Gerardo C. (2015). Malignant Triton tumors in sisters with clinical neurofibromatosis type 1. Case Rep. Oncol. Med..

[11] McConnell Y.J., Giacomantonio C.A. (2012). Malignant triton tumors—complete surgical resection and adjuvant radiotherapy associated with improved survival. J. Surg. Oncol..

[12] Tomaszewski Dariusz, Rogowski Jan (2017). Malignant triton tumor of lung, infiltrating the left atrium and left ventricle, with metastasis to small intestine. Kardiochir. Torakochirurgia Pol..

[13] Ducatman B.S., Scheithauer B.W. (1984). Malignant peripheral nerve sheath tumors with divergent differentiation. Cancer.

[14] Daimaru Y., Hashimoto H., Enjoji M. (1984). Malignant “triton” tumors: clinicopathologic and immunohistochemical study of nine cases. Hum. Pathol..

[15] Brooks J.S., Freeman M., Enterline H.T. (1985). Malignant Triton tumor. Natural history and immunohistochemistry of nine cases with literature review. Cancer.

[16] Wong S.Y., Teh M., Tan Y.O. (1991). Malignant glandular triton tumor. Cancer.

[17] Otani Y., Morishita Y., Yoshida I. (1996). A malignant Triton tumor in the anterior mediastinum requiring emergency surgery: report of a case. Surg. Today.

[18] Bose A.K., Deodhar A.P., Duncan A.J. (2002). Malignant triton tumor of the right vagus. Ann. Thorac. Surg..

[19] Lang-Lazdunski L., Pons F., Jancovici R. (2003). Malignant “Triton” tumor of the posterior mediastinum: prolonged survival after staged resection. Ann. Thorac. Surg..

[20] Loic Lang-Lazdunski M.D., Pons Francois, Rene Jancovici M.D. (2003). Malignant Triton Tumor of the posterior mediastinum: prolonged survival after staged resection. Ann. Thorac. Surg..

[21] Zisis C., Fragoulis S., Rontogianni D. (2006). Malignant triton tumor of the anterior mediastinum as incidental finding. Monaldi Arch. Chest Dis..

[22] Ren Wei, Xu Xinyun, Yan Jing (2014). Malignant triton tumor of the anterior mediastinum: a case report. Oncol. Lett..

[23] Stasik C.J., Tawfik O. (2006). Malignant peripheral nerve sheath tumor with rhabdomyosarcomatous differentiation (malignant triton tumor). Arch. Pathol. Lab. Med..

[24] Gupta G., Mammis A., Maniker A. (2008). Malignant peripheral nerve sheath tumors. Neurosurg Clin. N. Am..

[25] Terzic A., Bode B., Gratz K.W. (2009). Prognostic factors for the malignant triton tumor of the head and neck. Head Neck.

[26] Yakulis R., Manack L., Murphy A.I. (1996). Post radiation malignant triton tumor. A case report and review of the literature. Arch. Pathol. Lab. Med..

[27] Kamperis E., Barbetakis N., Asteriou C. (2013). Malignant triton tumor of the chest wall invading the lung. A case report and literature review. Hippokratia.

[28] Kroep J.R., Ouali M., Gelderblom H. (2011). First-line chemotherapy for malignant peripheral nerve sheath tumor (MPNST) versus other histological soft tissue sarcoma subtypes and as a prognostic factor for MPNST: an EORTC soft tissue and bone sarcoma group study. Ann. Oncol..

